# Malignant melanoma with synchronous thyroid metastases: case report and literature review

**DOI:** 10.1590/2359-3997000000251

**Published:** 2017-02-01

**Authors:** Maria Manuel Costa, Sandra Belo, João Capela-Costa, Jennifer Costa, Davide Carvalho

**Affiliations:** 1 Department of Endocrinology, Diabetes and Metabolism Faculty of Medicine University of Porto Centro Hospitalar São João Porto Portugal Department of Endocrinology, Diabetes and Metabolism, Faculty of Medicine University of Porto, Centro Hospitalar São João, Porto, Portugal; 2 Department of Surgery Faculty of Medicine University of Porto Centro Hospitalar São João Porto Portugal Endocrine Surgery Division, Department of Surgery, Faculty of Medicine University of Porto, Centro Hospitalar São João, Porto, Portugal; 3 Department of Pathology Centro Hospitalar São João Porto Portugal Department of Pathology, Centro Hospitalar São João, Porto, Portugal

## Abstract

Thyroid metastases are rare in clinical practice. We describe the case of an 85-year-old woman who was referred to our department due to a multinodular goiter with compressive symptoms and subclinical hyperthyroidism. The patient was also undergoing evaluation for a polyp in her left nasal cavity, which was then diagnosed as a malignant melanoma of the nasal mucosa. A thoracoabdominal magnetic resonance imaging obtained for cancer staging revealed a > 50% tracheal obstruction caused by the goiter. The patient underwent simultaneous total thyroidectomy and melanoma excision. Histological analysis of the thyroid showed the presence of multiple metastatic foci from the melanoma. Due to the patient’s age, a decision was made to maintain her under surveillance and administer palliative treatment if necessary. Although metastases to the thyroid are rare, they should be considered in the differential diagnosis of thyroid lesions in patients with a known primary tumor. The thyroidectomy, performed in this patient’s case, allowed the diagnosis of the metastases and relief of compressive symptoms caused by the goiter.

## INTRODUCTION

Metastases to the thyroid are rare, despite the gland’s rich vascular structure and blood supply (1). Some hypotheses proposed to explain the low rates of thyroid metastases include the fast blood flow within the gland, which would prevent adhesion of malignant cells, and its high concentration of oxygen and iodine, which inhibit the propagation and growth of malignant cells (2).

The frequency of thyroid metastases vary depending on the type of material analyzed. In autopsy series, the incidence of metastases to this gland varies from 1 to 24%, but only 1.4 to 3% of the patients undergoing surgery for thyroid malignancy present thyroid metastases (3-5). These different rates suggest that thyroid metastases are often occult (6). The most frequent primary tumors metastasizing to the thyroid are the lungs, the gastrointestinal system, the breast, and the kidneys (4,7,8).

Metastases may occur due to the spread of malignant cells via hematogenous or lymphatic dissemination or, alternatively, by a direct invasion of the thyroid by malignant neoplasms located in adjacent organs such as the larynx, tongue, esophagus, and proximal trachea (3). Malignant melanoma, an aggressive skin cancer with an increasing incidence, has the ability to metastasize to almost any organ, with the most common sites including the lungs, liver, and brain (9). Metastatic disease should always be considered in the differential diagnosis of patients with a history of cancer and presenting with thyroid nodules. Metastases are often found in patients with disseminated disease and are frequently a finding of a terminal disease (10).

Here, we describe the case of a patient with thyroid metastases from a nasal malignant melanoma and present a review of the literature on the topic of thyroid metastases.

## CASE REPORT

An 85-year-old Caucasian female was referred to our Endocrinology Department due to a thyroid goiter and subclinical thyrotoxicosis. She had known multinodular goiter for many years and had developed dyspnea, dysphagia, and hoarseness a few months before the referral. The patient had no prior history of cancer and no family history of thyroid disease.

On thyroid ultrasonography, the patient presented a multinodular goiter with the largest nodules measuring 35 mm and 21 mm, located in the left and right lobes, respectively. No cervical adenopathy was noted. Her thyroid function tests showed the following results: thyroid-stimulating hormone (TSH) 0.15 μU/mL (reference range 0.46-4.68), free triiodothyronine (T3) 4.15 pg/mL (2.75-5.26), and free thyroxine (T4) 1.07 ng/dL (0.77-2.19). Antithyroperoxidase and antithyroglobulin antibodies were negative.

The diagnosis of thyrotoxicosis was established, and therapy with methimazole 5 mg once daily was initiated due to the patient’s age and history of cardiovascular disease. Thyroid scintigraphy, cervical and chest X-ray, and measurement of thyrotropin-receptor antibodies were requested; however, the patient underwent surgery before undergoing these tests. Due to that, we were unable to clarify the etiology of her thyrotoxicosis, although, toxic multinodular goiter or damage to thyroid follicles by metastatic lesions seemed to be the two likely causes.

**Figure 1:**
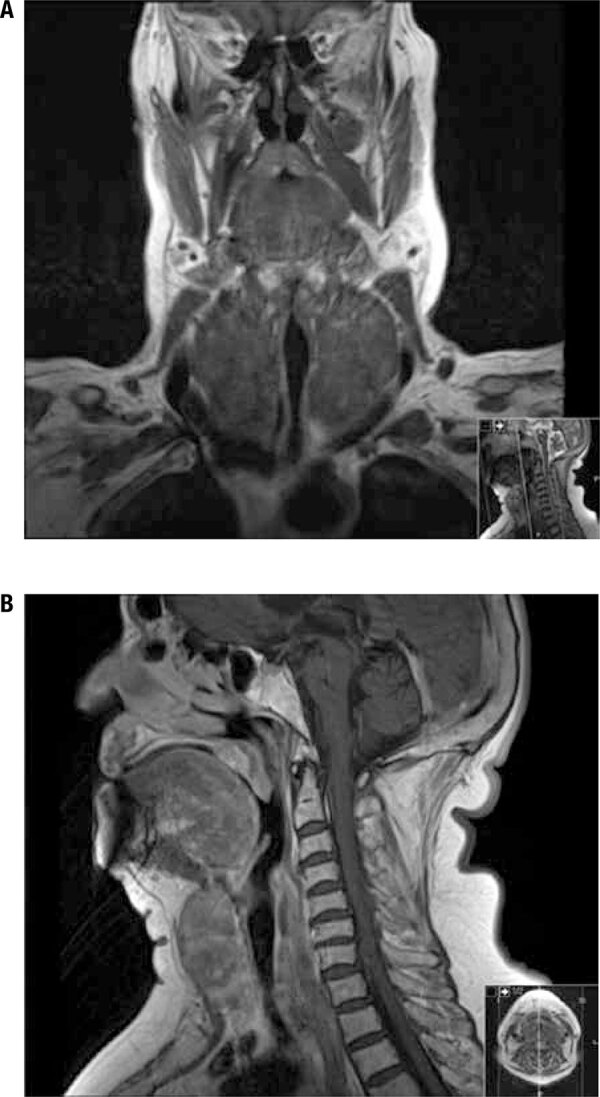
Magnetic resonance imaging of the head and neck showing compression of the trachea by a multinodular goiter. (A) Coronal view. (B) Sagittal view.

The patient was also undergoing an ear, nose, and throat (ENT) evaluation due to a polyp in her left nasal cavity measuring 41 x 29 mm, which occupied almost the entire cavity. The polyp was evaluated with fine-needle aspiration biopsy (FNAB), and the results suggested the diagnosis of malignant melanoma. For the purpose of cancer staging, she was investigated with thoracoabdominal magnetic resonance imaging which revealed a > 50% tracheal obstruction on the cervical-thoracic transition caused by her large multinodular goiter (Figure 1). No other lesions suggestive of metastatic disease were observed.

The patient subsequently underwent total thyroidectomy with left cervical lymph node dissection and removal of the melanoma from her nasal cavity. She was discharged from the hospital on the third postoperative day without any complications with a prescription of levothyroxine 100 μg/day.

**Figure 2:**
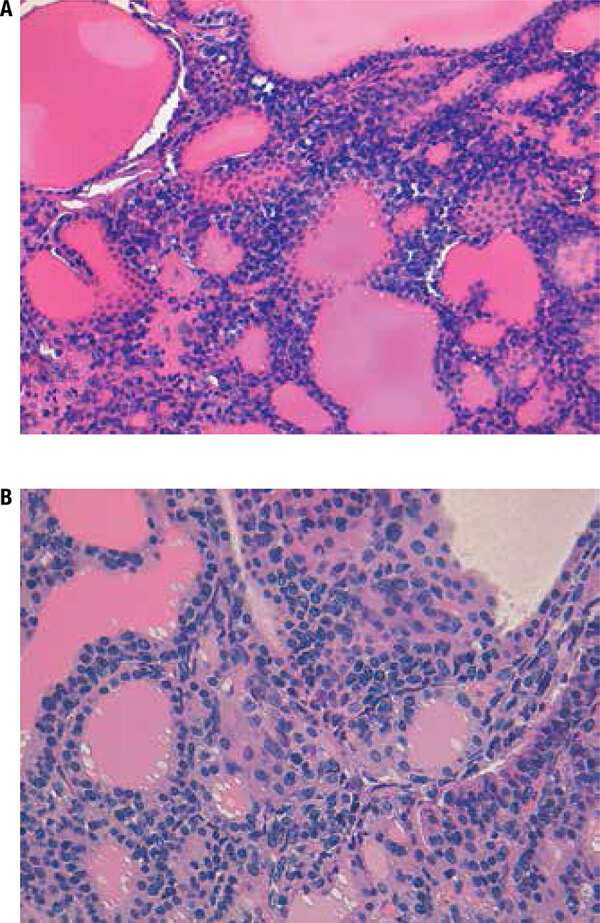
Thyroid histology showing (A) melanoma metastasis (H&E staining, x200) and (B) neoplastic cells infiltrating the parenchyma without destroying the thyroid follicles. Shown are epithelioid or spindle cells with granular chromatin and scant cytoplasm (H&E staining x 400).

**Figure 3:**
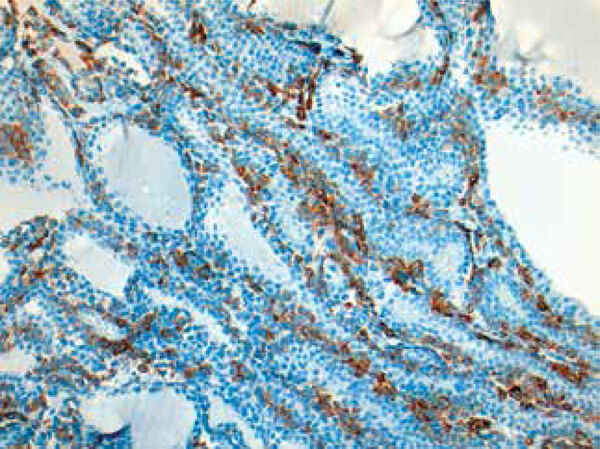
Histological analysis of the thyroid showing HMB-45 immunoexpression by the neoplastic cells (x200).

The histological report of the excised nasal polyp confirmed the diagnosis of malignant melanoma. On immunohistochemical staining, the tumor tissue showed diffuse immunoreactivity for HMB-45 and S-100 proteins. The histological analysis also revealed the presence of metastases in two out of 21 dissected lymph nodes. The histological analysis of the thyroid confirmed the diagnosis of a multinodular goiter with multiple bilateral cancer foci with overlapping characteristics to that of the nasal mucosa neoplasm (Figure 2) and immunoreactivity for HMB-45 (Figure 3).

Due to the patient’s age, a decision was made to maintain her under surveillance, with possible palliative treatment if necessary. Four months after surgery, the patient was hospitalized due to obstructive pyelonephritis and aggravation of a chronic renal disease. In this context, a computed tomographic (CT) scan of the abdomen and pelvis was performed, which revealed the presence of multiple lesions consistent with widespread metastatic disease. One month later, the patient presented a right femoral neck fracture in the absence of a fall or injury, which was interpreted as a pathologic fracture. She underwent surgical correction of the fracture but died two weeks later due to cardiorespiratory arrest.

## DISCUSSION

This case illustrates the presentation, diagnosis, and treatment of a patient with thyroid metastases. Thyroid metastases account for a low percentage of all malignant thyroid neoplasms. They occur most frequently between the sixth and seventh decades of life and have age as one of the main contributors of a poor prognosis (10,11). In the present case, the diagnosis was established at an even older age, at the ninth decade of life.

Primary tumors of the thyroid gland occur more frequently in women; however, in terms of thyroid metastases, some controversy exists regarding a gender predominance. Some studies suggest a higher incidence in females, while others report otherwise (4,12,13). According to their primary site, melanomas are grouped as cutaneous, ocular, mucosal, and of unknown origin; of all, mucosal melanomas are the least frequent ones (14). Head and neck mucosal melanomas (HNMMs) comprise 0.7% to 0.8% of all melanomas and less than 10% of all head and neck melanomas (15). Malignant melanoma is an aggressive cutaneous melanocytic neoplasm which often metastasizes to regional lymph nodes but whose mortality is mainly determined by tumor dissemination to visceral organs such as the lungs, liver, and brain. Although rare, HNMMs are very aggressive malignant tumors, and their prognosis is worse than that for cutaneous and ocular melanomas (16).

Metastatic melanoma to the thyroid is rare, with only a few cases described in the literature (17). In a recent meta-analysis that excluded thyroid metastases detected on autopsies, metastases of malignant melanomas accounted for 4% of 372 cases of thyroid metastases (4). Noncutaneous melanomas tend to present at an older age, as in the present case, and to be diagnosed at a more advanced stage (16,18).

Thyroid metastases may be the first finding in a tumor with an unknown location, leading to the diagnosis of the primary tumor, or may be a synchronous (20-40%) or metachronous (60-80%) manifestation of a known tumor (19). In the current case, the thyroid metastases were a synchronous finding. The moment when the thyroid metastases are diagnosed varies widely, ranging from the same time as the primary tumor (especially in the case of aggressive primary tumors) to up to 20 years after the primary tumor (in cases of less aggressive tumors) (13). This time gap may account for the difficulty of diagnosing these metastases (19). They can be solitary, multiple, or diffuse, and often present as multiple nodules, as in the present case (20).

Immunohistochemistry is a useful test to confirm the diagnosis of metastatic malignant melanoma. The S-100 protein is a sensitive but not very specific marker. More specific markers such as melan-A, HMB-45 and MITF are preferable in this regard (21).

The diagnosis of thyroid metastasis is often delayed since its signs and symptoms are usually mild, like the manifestations present in differentiated thyroid cancer. Affected patients may be asymptomatic and have a nodule detected during routine examination or while undergoing imaging tests to stage the primary tumor; they may also be symptomatic and report a new or enlarging thyroid nodule, enlarged thyroid gland, neck swelling, dysphagia, dysphonia, or cough, as observed in the present case report (4,13).

Most case reports of thyroid metastases omit the patient’s thyroid function; however, existing information in the literature report that most patients with thyroid metastases are euthyroid, although thyrotoxicosis or hypothyroidism may occur. Thyrotoxicosis, as observed in our patient, may be caused by invasion and damage to thyroid follicles by rapid growing metastases, with release of hormone into the bloodstream, or by the production of thyroid hormones by the tumor cells (13,22). Unfortunately, the study of the etiology of the thyrotoxicosis in our patient’s case was incomplete. Hypothyroidism can occur later in the context of parenchymal damage (4).

FNAB is a useful method to determine the differential diagnosis between benign and malignant thyroid lesions; however, the differentiation between primary and secondary malignant thyroid lesions is sometimes challenging and often achieved only after surgery. Despite the high sensitivity of FNAB for the diagnosis of metastases to the thyroid gland, the accuracy of FNAB is between 50% and 90% (19,23-25). This difference can be attributed to the experience of the center performing the FNAB (23). In cases of poorly differentiated tumors, immunohistochemistry may be required to determine their differential diagnosis, increasing the diagnostic accuracy and helping identify the primary neoplasm. Metastases fail to react to thyroglobulin, calcitonin, and thyroid transcription factor-1 (TTF-1) (26). It is important to highlight that anaplastic thyroid carcinomas also do not always react to thyroglobulin and 20-30% of the cases are negative on thyroid-specific immunohistochemistry (19,26). Our patient was not evaluated with thyroid FNAB because surgery had to be performed fast; her malignant melanoma had to be removed with some urgency, and due to her symptoms, it was decided that both surgeries should be performed simultaneously. The presence of thyroid metastases is associated with a poor prognosis, although the life expectancy depends mostly on the prognosis of the primary tumor, extent of the disseminated disease, and primary tumor stage, rather than in the dissemination of the primary tumor to the thyroid gland (16,27,28). Overall, 35-80% of the patients presenting with metastases to the thyroid have disseminated disease (4,29). Specifically regarding malignant melanomas, patients with metastatic melanoma are reported to have a 24-month survival regardless of the location of the metastases. The survival of our patient was below 8 months.

Due to the rare occurrence of thyroid metastases, their approach is based on data from retrospective reviews and isolated clinical reports; therefore, no consensus recommendations have been established in this regard (23). Treatment of thyroid metastases is primarily surgical, but the decision to operate on the patients will depend on their clinical condition, the primary site of the original tumor, presence of other metastases, the degree of dissemination, and symptoms caused by the thyroid mass (7,10,13). Surgery may also be important as a palliative treatment to relieve symptoms, particularly those associated with airway compression. Most experts agree that surgery is an appropriate approach to patients with resectable disease and an otherwise reasonable prognosis (19). No consensus exists on the extent of the surgery, and most authors recommend that isthmectomy and lobectomy be performed in cases with isolated nodules, whereas total or near-total thyroidectomy should be performed in cases with multifocal disease (13). In the present case, the patient had a multinodular goiter with compressive symptoms, which was the reason for her undergoing total thyroidectomy.

Although some studies suggest that surgical treatment extends the survival of patients with thyroid metastases, the evidence in this regard remains inconclusive (4,13). The factors associated with a better prognosis in patients undergoing surgery are the absence of metastases to other sites, renal carcinoma as the primary tumor, and a long period between the diagnosis and the development of the primary tumor (19). In cases of patients with disseminated disease or comorbidities contraindicating surgery, radiotherapy and chemotherapy have been used as palliative treatments (19,28). Since there have been only a few reports of melanomas metastatic to the thyroid, the role of surgery in this aggressive malignancy has not yet been well defined; however, considering the aggressiveness of this tumor, the role of surgery seems to be limited to the relief of compressive symptoms rather than cure (30).

## CONCLUSION

We described a rare case of thyroid metastases from malignant melanoma to draw attention to the differential diagnosis of thyroid lesions. During the evaluation of a thyroid nodule, particularly in patients with a history of malignancy, the hypothesis of metastases should always be considered.
